# Test of Pure Air

**Published:** 1873-10

**Authors:** 


### TEST OF PURE AIR.

Dr. Angus Smith gives a good rule for ascertaining the amount of carbonic acid in the air of houses: "Let us keep our rooms so that the air does not give a precipitate when a 10½ ounce bottleful is shaken with half an ounce of clear lime-water;" a sanitary regulation which is easily carried out.—*Med. & Sur. Reporter.*

